# Comment on “Relation of fruit juice with adiposity and diabetes depends on how fruit juice is defined: a re-analysis of the EFSA draft scientific opinion on the tolerable upper intake level for dietary sugars” by Chen et al. 2023

**DOI:** 10.1038/s41430-023-01337-0

**Published:** 2023-09-15

**Authors:** Silvia Valtueña Martínez, Dominique Turck, Ionut Craciun, Marco Vinceti

**Affiliations:** 1 Senior Scientific Officer, European Food Safety Authority, Parma, Italy; 2 Chair of the Panel on Nutrition, Novel Foods and Food Allergens (NDA), European Food Safety Authority, Parma, Italy; 3 Scientific Officer, European Food Safety Authority, Parma, Italy; 4 Chair of the Working Group on Sugars and member of the NDA Panel, European Food Safety Authority, Parma, Italy

**Keywords:** Obesity, Nutrition

**To the Editor:**

The article by Chen and colleagues [1] provides a re-analysis of the relationship between fruit juices (FJ) and risk of obesity and type 2 diabetes based on EFSA’s draft Scientific Opinion on the Tolerable Upper Intake Level for dietary sugars. The draft opinion was made available for public consultation between July and September 2021 to collect comments from the scientific community and other stakeholders. The draft was therefore provisional and did not include subsequent revisions published in February 2022’s final version [2]. This is regrettable as the final version comprehensively addressed the premise of Chen et al.’s article.

The meta-analyses and dose-response analysis on FJ and risk of type 2 diabetes that are now published by Chen and colleagues were submitted by the European Fruit Juice Association during the public consultation in 2021 (comment 265 in the technical report of the public consultation, Annex O) [2]. Stakeholders also raised the issue that FJ consumption could have been misclassified in prospective cohort studies (PCs), and that the EPIC-InterAct study [3] was mistakenly excluded by EFSA during the screening phase. Based on these comments, EFSA’s final opinion concludes on FJ in general (and not on 100% FJ in particular) and includes the EPIC-InterAct study [3] replacing the EPIC-E3N [4] and EPIC-Norfolk [5] cohorts because of duplicate data. Therefore, the analyses by Chen et al. do not reflect the totality of the evidence available to EFSA for the final opinion.

Chen and colleagues mention that EFSA assessors did not quantify the relationship between 100% FJ intake and adiposity outcomes and used instead vote counting, which they consider an invalid approach for evidence synthesis. Besides the fact that the conclusion of the final version of the opinion [2] again refers to FJ in general, they fail to mention the heterogeneity of the studies available regarding the study population (adults, children) and endpoints measured: i) obesity as dichotomous endpoint, ii) 4-y change in body weight, iii) change in body weight for the study period, iv) change in BMI, v) 1-y change in BMI z-scores, vi) change in BMI z-scores over the study period. This precluded quantitative data synthesis, as discussed in the comprehensive uncertainty analysis conducted by EFSA on the body of evidence (BoE).

Chen et al. pool data from 2 PCs on risk of abdominal obesity, from 4 PCs on body weight, and from 4 PCs on BMI z-scores, separately. EFSA concluded that the available BoE does not suggest a positive relationship between the intake of FJs and risk of abdominal obesity based on the 2 PCs available. A quantitative synthesis of the evidence was not needed to reach this conclusion, neither justified, considering the low number of studies available. Regarding the risk of obesity, Chen et al. conclude that consumption of 100% FJ was associated with an increase in BMI z-scores in children (4 PCs) and an increase in body weight in adults (4 PCs), although they question the clinical relevance of the findings, based on a quantitative synthesis of the evidence. EFSA concluded that the available BoE, which includes the 8 PCs, suggested a positive relationship between the intake of FJs and risk of obesity based on the direction of the association between the exposure and the endpoint. The level of certainty on the causality of the association was considered very low (0–15%) owing to the limitations in the BoE, including the internal validity of the studies (risk of bias) and the use of surrogate endpoints. Again, a quantitative evidence synthesis was considered inappropriate because of the heterogeneity of the endpoints measured in the studies.

All considered, it is unfortunate that Chen et al.’s article, already outdated at the time of publication, does not acknowledge EFSA’s final opinion [2].

## Data Availability

Data sharing not applicable as no datasets were generated or analysed for this comment.
