# Correction: Comment on “Relation of fruit juice with adiposity and diabetes depends on how fruit juice is defined: a re-analysis of the EFSA draft scientific opinion on the tolerable upper intake level for dietary sugars” by Chen et al. 2023

**DOI:** 10.1038/s41430-023-01388-3

**Published:** 2023-12-15

**Authors:** Silvia Valtueña Martínez, Dominique Turck, Ionut Craciun, Marco Vinceti

**Affiliations:** 1 Senior Scientific Officer, European Food Safety Authority, Parma, Italy; 2 Chair of the Panel on Nutrition, Novel Foods and Food Allergens (NDA), European Food Safety Authority, Parma, Italy; 3 Scientific Officer, European Food Safety Authority, Parma, Italy; 4 Chair of the Working Group on Sugars and member of the NDA Panel, European Food Safety Authority, Parma, Italy

**Keywords:** Obesity, Nutrition

Correction to: *European Journal of Clinical Nutrition*

10.1038/s41430-023-01337-0, published online 15 September 2023

The following disclaimer was added in the article backmatter:

“The authors Silvia VALTUEÑA MARTINEZ and Ionut CRACIUN are employed with the European Food Safety Authority (EFSA) in NIF Unit that provides scientific and administrative support to the EFSA NDA Panel in the area of nutrition. However, the present article is published under the sole responsibility of the authors and may not be considered as an EFSA scientific output. The positions and opinions presented in this article are those of the authors alone and do not necessarily represent any official position of EFSA. To know about the views or scientific outputs of EFSA, please consult its website under http://www.efsa.europa.eu.” The original article has been corrected.

